# Exploring the Treatment Journey of People Living With HIV in Iran: A Qualitative Study

**DOI:** 10.1155/arat/9983262

**Published:** 2026-06-07

**Authors:** Asma Baniasad, Ghoncheh Raheb, Kian Norouzi Tabrizi, Maryam Latifian

**Affiliations:** ^1^ Department of Social Work, Substance Abuse and Dependence Research Center, University of Social Welfare and Rehabilitation Sciences, Tehran, Iran, uswr.ac.ir; ^2^ Substance Abuse and Dependence Research Center, University of Social Welfare and Rehabilitation Sciences, Tehran, Iran, uswr.ac.ir; ^3^ Department of Nursing, University of Social Welfare and Rehabilitation Sciences, Tehran, Iran, uswr.ac.ir

**Keywords:** experience, Iran, PLHIV, qualitative research, treatment

## Abstract

**Introduction:**

Today, HIV infection is considered among the most important infectious diseases worldwide. This study aimed to explore the challenges of people living with HIV (PLHIV) on treatment journey.

**Methods:**

This study was conducted using a qualitative approach and the content analysis method at the Behavioral Diseases Consultation Center and the Positive Club of Kerman, Iran, from September 2023 to August 2024. A total of 27 individuals, including 17 PLHIV, 5 caregivers, and 5 social work and HIV professionals, were recruited using purposive sampling. Data were collected using individual semistructured interviews, field attendance, and note‐taking.

**Results:**

Data analysis resulted in 1258 primary codes, which were categorized with the theme of challenges of living with HIV into 30 subcategories and 7 main categories, including emotional and psychological tensions, objective and behavioral reflections, social deprivation, unfavorable social policies, changes in life, labeling, poor quality, and insufficient medical services.

**Conclusion:**

Due to the numerous problems and challenges faced by PLHIV during their treatment, they need comprehensive support from their families, caregivers, and society. Ensuring access to appropriate medical services and enhancing public awareness and understanding of PLHIV and their caregivers through education are essential. Such measures can contribute to improved treatment adherence, better quality of life, and more effective patient‐centered care for PLHIV.

## 1. Introduction

As the causative agent of AIDS, HIV poses one of the most serious public health challenges globally. There is currently a global commitment to prevent the emergence of new HIV infections and to ensure the accessibility of treatment for all PLHIV [1]. Although the clinical aspect of AIDS has received more recognition so far, its cultural, social, and economic dimensions are also important and deterministic in its incidence rate, causing various health, social, cultural, and economic consequences at the community level [2]. According to the Joint United Nations Programme on HIV/AIDS (UNAIDS), in 2024, an estimated 40.8 million people were living with HIV globally, and 630,000 people died of AIDS‐related illnesses. In Iran, the number of PLHIV has been reported to be 53,000, of whom 3500 died in 2021 [3].

The most important disease under care among communicable diseases in Iran in the last 2 decades is HIV/AIDS. The cost of HIV/AIDS care in Iran is equal to that of all other infectious diseases. Despite the extensiveness of the care system in Iran, perhaps the most important reason for the low sensitivity of the care system is the stigma attached to patients, which has caused a large number of PLHIV to go undiagnosed [4]. Due to adverse social consequences caused by prevailing social beliefs and social stigma, HIV/AIDS is considered a disease with profound impacts not only on the physical health of patients but also on their social and mental well‐being [5].

Inadequate knowledge among at‐risk populations and PLHIV, along with incorrect social beliefs, particularly regarding transmission routes can intensify stigmatization. Fear of infection through routine social interactions often leads others to avoid contact with PLHIV, resulting in social isolation and loss of self‐confidence over time [6]. Moreover, high‐risk asymptomatic individuals are widely distributed across the country and are difficult to identify. These people often struggle to communicate with healthcare providers due to social, cultural, and economic barriers [7]. Although HIV is a global epidemic, the lived experiences of PLHIV differ across countries depending on cultural norms, social attitudes, healthcare policies, and available support systems.

Several studies have explored different aspects of HIV/AIDS in Iran, particularly in Kerman city. However, many of these studies have given limited attention to the challenges faced by PLHIV, particularly during the treatment process. Given the complex and multifaceted nature of HIV/AIDS, understanding patients’ experiences is essential for improving patient‐centered care and supportive interventions. Therefore, this study aimed to explore the experiences and challenges of PLHIV in Iran during the course of treatment. In this study, the treatment process refers primarily to long‐term antiretroviral therapy (ART), including regular medication use, clinical follow‐up, and coping with treatment‐related physical, psychological, and social challenges, in order to provide evidence that may inform healthcare providers, policymakers, and family members in supporting PLHIV more effectively.

## 2. Methods

### 2.1. Study Design

This qualitative study was conducted using the content analysis method. Content analysis is a research method for the mental interpretation of textual data, through which obvious and hidden themes or patterns are identified using a regular classification process [8]. It is necessary to mention, according to the purpose, the present research is part of exploratory studies, and according to the results, it is part of applied research.

### 2.2. Participants

The research participants included PLHIV, their caregivers, and social workers or HIV professionals. Participants were selected using purposive sampling. A total of 27 participants were interviewed; data saturation was achieved after 27 interviews, as no new codes emerged.

Inclusion criteria for PLHIV were age between 18 and 65 years and a confirmed HIV diagnosis by a physician based on standard diagnostic tests. Caregivers had to be primary family members of people with HIV, living with and caring for them. Additionally, inclusion criteria for experts included having more than 5 years of experience in palliative care or HIV‐related services or research experience in these fields, as well as holding at least a bachelor’s degree. Exclusion criteria for all participants were that they did not have enough mental ability to participate in the research or that they lost the desire to continue participating in the study.

### 2.3. Data Collection

The study was conducted at the HIV Voluntary Counseling and Testing Center (VCT‐HIV) and the Positive Club in Kerman, where PLHIV regularly receive services. Data collection was conducted in the winter of 2023. First, using the literature review, an overview of the concepts of doing and the framework of the questions were identified. Eligible participants were referred to the researchers by center staff. The researchers explained the study to these individuals and invited them to participate. Written informed consent was then obtained from all participants prior to their involvement in the study. Participants were fully informed about the study objectives, procedures, and their right to withdraw at any time, and confidentiality of all personal data was strictly maintained.

In the next step, the necessary information was collected through in‐depth and semi‐structured interviews (in person interviews lasting 30–90 min) and field notes. Based on the instructions and the guide, each interview began with an open‐ended question, followed by probing questions aligned with the study objectives. The initial open‐ended question was: “please talk about your experiences of the HIV treatment process.” Probing questions followed to explore participant’s responses in depth.

### 2.4. Data Analysis

Qualitative content analysis was conducted using the approach proposed by Graneheim and Lundman [9]. For this purpose, in the first step, a review of the interviews, general understanding, and identification of the main concepts was done.

Texts of the interviews were broken down into constituent semantic units and then into the smallest meaningful units after reviewing it several times. In the next step, codes were read several times and based on the semantic similarity; they were placed in subcategories and categories. Initial coding was performed independently by two researchers, with all authors involved in finalizing categorization. The final categories were reviewed repeatedly by all the authors, until finally the researchers reached an agreement on the main categories. Data analysis was performed during data collection, and before conducting each interview, the previous interview was rewritten and analyzed. Sampling and data analysis lasted one year (September 2023 to August 2024). In order to coding, MAXQDA 2020 software was used.

### 2.5. The Study Rigor

Rigor of the study was established based on the criteria proposed by Lincoln and Guba and Lincoln [10] and Cardano [11]. To enhance credibility, maximum variation sampling was employed, and participants were selected to ensure diversity in demographic characteristics, including age, marital status, education, and employment status, in order to capture a wide range of perspectives. Triangulation of data sources was also used, and information was received from three different sources: PLHIV, caregivers, and professionals. To achieve dependability, the findings were shared with a few participants for conduct member checking, and all participants confirmed the interpretations. To promote confirmability, while discarding the researcher biases, the evaluation and coding procedures were shared among all authors and expressed their opinions.

To enhance transferability, a thorough explanation of the whole research process was provided, and the participants’ conversations were described directly and a clear description of the context, selection and characteristics of the participants, data collection, and analysis process is provided so that the reader can judge the applicability of the findings in other situations. Table 1 presents an example of the coding and classifying concepts.

**TABLE 1:** An example of the coding process (social deprivation).

Text unit	Code	Subcategory	Category
“My mother‐in‐law came to the hospital for an x‐ray and saw me working there. She told the hospital president and administrative manager that I had AIDS. The president called me and, although I explained the precautions I take at work, he said I could injure myself while handling files. After that, he transferred me to another position.”	Loss of job position	Occupational challenges	Social deprivation
“Marital life requires long‐term goals, such as building a future, having children, or companionship in old age. Because my future feels uncertain, I do not want any of these, and therefore I have decided not to marry.”	Decision not to marry	Threat of marriage opportunities	
“Unlike cancer patients, I face restrictions on travel. I previously had to falsify documents in order to travel to Russia. When I returned to Iran for my child’s birth and needed special medical care, this could no longer be concealed. As a result, I lost the opportunity to obtain a Russian visa for my son.”	Inability to migrate	Loss (opportunities and relationships)	

### 2.6. Ethical Considerations

Written informed consent was obtained from all participants. It was clarified to them that there is no obligation to participate in the research and they could withdraw from the interview at any time without any consequences. Participants were also assured about the method of publishing the results and the confidentiality of the information.

## 3. Results

A total of 27 participants were included in this study, comprising 17 PLHIV, 5 caregivers of PLHIV, and 5 professionals in the fields of social work and HIV. The characteristics of the participants in the research are mentioned in Table 2. Regarding caregivers of PLHIV, the mean and standard deviation of age was (30 ± 9.93). Regarding their relationship to PLHIV, 40% were spouses, 20% mothers, 20% children, and 20% sisters. Forty percent of caregivers had completed middle school, 40% had a diploma, and 20% held an associate degree. For PLHIV, the mean and standard deviation of age (43.35 ± 8.10) and the mean time since diagnosis was 8.35 ± 5.38 years. 70.58% of participants were female, and 29.41% were male. Educational levels included elementary school (35.29%), middle school (11.76%), diploma (29.41%), and bachelor’s degree (17.64%). Regarding marital status, 11.76% were single, 52.94% married, 5.88% divorced, and 29.41% widowed. The five experts included three social workers (two with bachelor’s degrees and one with a master’s degree), one counselor with a master’s degree in clinical psychology, and one general practitioner. The mean and standard deviation of their age (42.8 ± 3.96) and the mean and standard deviation of their activity in social work and HIV (4.30 ± 13.8) were calculated.

**TABLE 2:** Characteristics of participants in the research.

PLHIV						
Number of family members	Duration of diagnosis (years)	Occupation	Education	Marital status	Age	Gender
2	4	Embroidery	Elementary school	Divorced	48	Female
3	10	Embroidery	Elementary school	Widow	47	Female
6	7	Unemployed	Diploma	Married	32	Female
4	5	Unemployed	Elementary school	Widow	52	Female
3	22	Retired	Middle school	Widow	51	Female
3	1	Unemployed	Diploma	Married	32	Female
5	15	Unemployed	Elementary school	Married	49	Female
4	15	Social worker	Bachelor	Married	42	Female
5	8	Unemployed	Elementary school	Widow	55	Female
2	15	Unemployed	Elementary school	Widow	49	Female
2	7	Pistachio business	Bachelor	Married	34	Female
3	6	Unemployed	Diploma	Married	35	Female
4	10	Driver	Diploma	Married	48	Male
2	5	Club manager	Diploma	Married	30	Male
1	6	Employee	Bachelor	Single	36	Male
2	4	Pensioner	Diploma	Married	52	Male
1	2	Waste collection	Middle school	Single	45	Male

**Caregiver of PLHIV**						
**Family member’s illness duration (years)**		**Occupation**	**Education**	**Relationship with patient**	**Age**	**Gender**

8		Self‐employed	Diploma	Spouse	45	Male
2		Employee	Associate degree	Child	21	Female
8		Housewife	Diploma	Spouse	42	Female
19		Housewife	Middle school	Sister	30	Female
21		Housewife	Diploma	Mother	47	Female

**Social work and HIV experts**						
**Duration of activity in the field of social work or HIV (years)**		**Occupation**	**Expertise**		**Age**	**Gender**

12		Bachelor’s	Social worker		42	Female
11		Bachelor’s	Social worker		48	Female
10		Masters	Social worker		36	Male
14		Masters	Counselor		43	Female
22		General practitioner	General practitioner		45	Male

**FIGURE 1:**
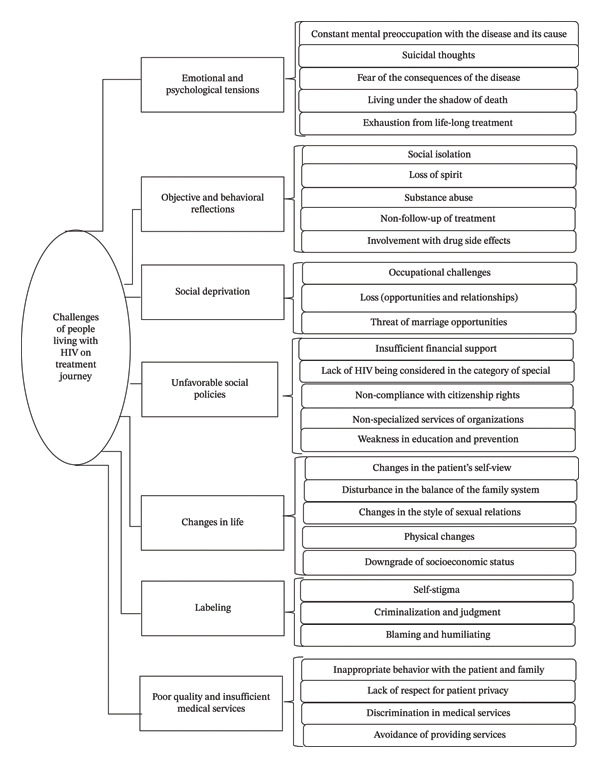
Introduction of subcategories, categories, and of their research.

Data analysis resulted in 1258 primary codes, which were organized into 30 subcategories and 7 main categories. In the following, each of the codes will be explained, and several quotes from the participants will be mentioned. Concepts, categories, and subcategories are summarized in Figure 1.

### 3.1. Emotional and Psychological Tensions

The concept of emotional and psychological tensions was identified as one of the most important concepts. Constant mental preoccupation with the disease and its cause, suicidal thoughts, fear of consequence of the disease, living under the shadow of death, and exhaustion from life‐long treatment are included in this category.

#### 3.1.1. Constant Mental Preoccupation With the Disease and Its Cause Was Reported in 13 Interviews

“You cannot find a night in which I could sleep well unless I take sleeping pills and painkillers. These thoughts are driving me crazy. I laugh and talk in front of my wife and children, but my heart is torn apart. There is no day I forget to think about it.”

#### 3.1.2. Suicidal Thoughts Were Reported by 8 Participants

“When my test came back positive, I thought I would die soon and leave my children without a mother. I was all alone and overwhelmed by sadness. That night, I considered ending my life, but my belief in God prevented me.”

#### 3.1.3. Fear of the Consequences of the Disease, Including Fear of Disclosure, the Future, and Rejection, Was Mentioned by 18 Participants

“My daughter’s morals are different from my son’s; she only thinks about her, has many needs, and wants to dress like her friends. Can I tell her that not only can I not afford her demands, but I also have AIDS? What will happen to my child?”

#### 3.1.4. Living Under the Shadow of Death Was Reported by 8 Participants

“I had heard that PLHIV live no more than ten years. I used to write in a notebook at night or during the day, I remember writing why? What will happen in the next 10 years? What will happen to my children?”

#### 3.1.5. Exhaustion From Life‐Long Treatment Was Mentioned by 6 Participants

“It is clear that she is exhausted from the inside—from these medications, from commuting to these centers. Why should anyone have to take pills for the rest of their life?”

### 3.2. Objective and Behavioral Reflections

The concept of objective and behavioral reflections was identified as another concept, and social isolation, loss of spirit, substance abuse, non‐follow‐up of treatment, and involvement with drug side effects are included in this category.

#### 3.2.1. Social Isolation Was Mentioned by 9 Participants

“He stays at home most of the time. Only in emergencies do we go out together, for example, to buy medication. He even begs me to go and buy the medicines all by myself, but I insist he should come to at least have a doctor′s visit.”

#### 3.2.2. Loss of Spirit Was Also Mentioned in 6 Interviews

“Well, psychologically, I am devastated; I constantly cry; in my loneliness, I cry; my daughter says to me that she has been exhausted seeing me always crying.”

#### 3.2.3. Substance Abuse Was Mentioned in 2 Interviews

“Since I realized I have the disease, I have doubled my substance use; it comforts me and makes me forget about my illness temporarily.”

#### 3.2.4. Non‐Follow‐Up Treatment Was Mentioned in 3 Interviews

“We are all simmered with anger with him. Sometimes he becomes stubborn or lazy; he should be forced to take his medications. He says, ‘So what if I die? What would happen? You don’t even care if I live or die.”

#### 3.2.5. Involvement With Drug Side Effects Was Proposed by 6 Participants

“These pills make me sick most of the time. During my pregnancy, my memory waned, my hands become frequently numb, and I have to inject vitamin B12 and Neurobion. I always carry a multivitamin pill with me.”

### 3.3. Social Deprivation

This category included occupational challenges, threat of marriage opportunities, and loss (opportunities and relationships).

#### 3.3.1. Occupational Challenges Were Reported by 6 Participants

“My mother‐in‐law came to the hospital for an x‐ray and saw me working there. She told the hospital president and administrative manager that I had AIDS. The president called me and, although I explained the precautions I take at work, he said I could injure myself while handling files. After that, he transferred me to another position.”

#### 3.3.2. Threat of Marriage Opportunities Was Reported by 9 Participants

“Marital life requires long‐term goals, such as building a future, having children, or companionship in old age. Because my future feels uncertain, I do not want any of these, and therefore I have decided not to marry.”

#### 3.3.3. Loss (Opportunities and Relationships) Was Reported by 5 Participants

“Unlike cancer patients, I face restrictions on travel. I previously had to falsify documents in order to travel to Russia. When I returned to Iran for my child’s birth and needed special medical care, this could no longer be concealed. As a result, I lost the opportunity to obtain a Russian visa for my son.”

### 3.4. Unfavorable Social Policies

Unfavorable social policies are among the other concepts identified, insufficient financial support and lack of HIV being considered in the category of special and incurable patients, noncompliance with citizenship rights, nonspecialized services of organizations, and weakness in education and prevention.

#### 3.4.1. Insufficient Financial Support Was Mentioned by 6 Participants

“Due to the medications consumed by patients, they need vitamins and supplements, which are not free of charge. I have frequently commuted to the welfare organizations and medical centers, but they told me that the free therapeutic care and services do not cover these supplements.”

#### 3.4.2. Lack of HIV Being Considered in the Category of Special and Incurable Patients Was Also an Inappropriate Policy That 3 Participants Mentioned

“MS patients can access loans easily, but we cannot, despite higher expenses, because we receive free treatment and care services.”

#### 3.4.3. Noncompliance With Citizenship Rights Was Discussed by 2 Participants

“According to the rights of citizenship, everyone is equal. But As a patient, I face discrimination everywhere. For example, a group of patients can receive certain services, they can receive citizenship, but I can’t travel easily; I can’t get a government job.”

#### 3.4.4. Nonspecialized Services of Organizations Were Reported by 4 Participants

“The wife of a patient with HIV and with spinal cord injury: she is under the coverage of the welfare organization. The welfare organization is aware of her situation; the welfare organization does well, but its services are only financial and do not provide psychological support.”

#### 3.4.5. Weakness in Education and Prevention Was Mentioned by 7 Participants

“The government has the responsibility to educate people and instruct them not to go toward wrongdoings. Why one should contract the virus. In my opinion, most blame lies with the government, not the affected individuals.”

### 3.5. Changes in Life

Changes in life are another identified concept. Changes in the patient’s self‐view, disturbance in the balance of the family system, changes in the style of sexual relations, physical changes, and downgrade of socioeconomic status are included under this concept.

#### 3.5.1. Changes in the Patient’s Self‐View Were One of the Changes Mentioned by 5 Participants

“This was like a storm devastating the lives of not only mine but also my sisters and brothers. This disease destroys everything; it destroys life. You cannot be the same person as before, and this feeling is shared by you and those around you. If this is not a storm, then what is it?”

#### 3.5.2. Disturbance in the Balance of the Family System Was Reported by 11 Participants

“My husband’s condition improved, but I cannot trust him anymore. We share a roof, but I do not have affection for him or even the desire to talk to him. He is the one to blame for all these problems. I wish he would say I’m sorry.”

#### 3.5.3. Changes in the Style of Sexual Relations Were Reported by 5 Participants

A spouse of PLHIV: “During the 7 years when she was sick, but we were not aware of this, we had a better relationship. Since the diagnosis was made, our communication has decreased a lot. When there is fear in everything you don’t enjoy anything.”

#### 3.5.4. Physical Changes Were Mentioned by 3 Participants

“Another issue that bothered me was that I lost weight. I can show you my picture. I was obese. I feel like I have lost my beauty.”

#### 3.5.5. Downgrade of Socioeconomic Status Was One of the Issues Mentioned by 8 Participants

“When my husband became ill, he was debilitated within not more than six months. I sold my car; I believed that he would recover, but I went through grave financial problems. At the very first, financial problems devastated us.”

### 3.6. Labeling

Labeling is another concept identified in this research. Self‐stigma, criminalization and judgment, blaming, and humiliation fall under this category.

#### 3.6.1. Self‐Stigma Was Raised by 8 Participants

“When I hug my child, seeing him turn his head toward my breast makes me loathe myself. I always wish my son would forgive me; I always feel that I owe him; I feel guilty about this.”

#### 3.6.2. Criminalization and Judgment Were Discussed by 13 Participants

“When the issue of AIDS comes up, everyone thinks that the affected person is an addict or a pervert, but I was innocent, and many of those that I know were innocent.”

#### 3.6.3. Blaming and Humiliating Were Mentioned in the Conversations of 7 Participants

“I blamed my father. Why does such an event have to happen? Why would you do such a thing that the child feels like this? Look what disaster you brought to my mother.”

### 3.7. Poor Quality and Insufficient Medical Services

This category was another concept identified. Inappropriate behavior with the patient and family, lack of respect for patient privacy, discrimination in medical services and avoidance of providing services are included in this category.

#### 3.7.1. Inappropriate Behavior With the Patient and Family Was Raised by 7 Participants

“My daughter admitted to the hospital. I talked to her infectious disease doctor, and she told me that he would speak to her doctor because I told him that I was afraid and anxious to disclose. As soon as she was discharged, my phone rang; it was a nurse shouting at me, insulting me, asking why I did not tell the truth.”

#### 3.7.2. Lack of Respect for Patient Privacy Was Discussed in 7 Interviews

“Such destructive behaviors have always been around. Recently, I became sick and admitted to a hospital. My problem was written above my bed, and everyone was told about this and warned not to approach me. It was complicated for me, and it was expensive.”

#### 3.7.3. Discrimination in Medical Services Was Mentioned by 2 Participants

“When someone gets sick, others treat them normally. But for us, they show different attitudes and become frightened.”

#### 3.7.4. Avoidance of Providing Services Was One of the Common Cases Mentioned in 15 Interviews

“The staff of the hospital would act very strangely. For example, I had tuberculosis, and they wanted to take me for lung sampling. In the operating room, all the nurses were wearing face masks, and the doctor refused to operate on me. I was crying there, and I was very agitated. I promised I would never ever put my foot in that hospital.”

## 4. Discussion

This study highlighted the multidimensional challenges faced by PLHIV in Iran across emotional, behavioral, social, and structural domains. To better contextualize these findings, we interpreted the results through the Information‐Motivation‐Behavioral Skills (IMB) model, which provides a framework for understanding health‐related behaviors, including treatment adherence.

The findings of this study indicate that PLHIV experience substantial emotional and psychological tension, including suicidal thoughts, constant mental preoccupation with the disease and its cause, fear of consequence of the disease, living under the shadow of death, and exhaustion from life‐long treatment. These challenges are closely linked to the motivation component of the IMB model. Inadequate emotional support and information reduce motivation to engage with care and adhere to treatment. Knowledge gaps and misconceptions observed among PLHIV and their families are directly related to the Information component. Misunderstandings about transmission, treatment, and disease progression contribute to fear, stigma, and inappropriate coping strategies. Improving patient education and public awareness can enhance information, thereby supporting better engagement with care. These finding are consistent with Camellia [12], Safarzadeh Jahromy et al. [13], and Winston and Spudich [14], who reported higher rates of mental health problems and cognitive impairments among PLHIV globally.

Objective and behavioral reflections, including social isolation, loss of spirit, substance abuse, non‐follow‐up treatment, and involvement with drug side effects, were among the findings of this research that correspond to the behavioral skills dimension of the IMB model. Patients may lack the skills or strategies to manage side effects, communicate with healthcare providers, or navigate social challenges, resulting in nonadherence and social withdrawal, which was in line with the report of Fallahi [15], who identified drug side effects as a physical outcome and isolation as a psychological consequence of living with HIV. de Los Rios et al. [16] noted that stress and difficulties caused by everyday medication and the fear of daily drug use disclosing their condition were among the physical, emotional, and psychosocial challenges related to daily medication dosing. In line with our results, Sana Ullah [17] highlighted that negative attitudes toward PLHIV would cause a sense of fear, isolation, deprivation, and treatment withdrawal.

Social deprivation, encompassing occupational challenges, threat of marriage opportunities, loss of opportunities, and relationships were other challenges identified. In parallel with these findings, Fallahi [15] declared social consequences as limitations in social, occupational, and marital opportunities. Also, D’Cruz [18] stated that HIV infection could lead to various types of discrimination in the workplace, such as denial of promotion, rejection, isolation, and abuse. According to the results of Anglewicz and Reniers [19], PLHIVs are more likely to have their marriage dismantled (either divorced or widowhood) and have lower chances of remarriage. The sociocultural context of Iran further shapes these experiences. Cultural norms surrounding marriage, family responsibilities, and gender roles intensify stigma, particularly for women, limiting their social participation and disclosure of their HIV status. Social deprivation reflects how structural and cultural barriers constrain patients’ behavioral skills and motivation. The present study highlighted unfavorable social policies, such as lack of HIV being considered in the category of special and incurable patients, noncompliance with citizenship rights, nonspecialized services of organizations, weakness in education, and prevention, which were in line with the results of Khodayari Zarnaq [20].

Unfavorable social policies and insufficient healthcare support also impede patients’ behavioral skills and motivation. The lack of integration of mental health services, inadequate financial and social support, and absence of specialized care programs for PLHIV limit patients’ ability to adhere to treatment and maintain quality of life. Furthermore, poor quality and insufficient medical services, including breaches of confidentiality and discriminatory practices, exacerbate these challenges.

According to the results of the present study, changes in life included changes in the patient’s self‐view, disturbance in the balance of the family system, changes in the style of sexual relations, physical changes, and downgrade of socioeconomic status, which was in line with the results of Dejman [5], reporting familial conflicts, unemployment, the need for housing, and basic life requirements among the problems experienced by PLHIV. In agreement, Bor et al. [21, 22] explored the impact of HIV on familial affairs, social stigma, isolation, secrecy, and stress, denoting that regardless of the route of transmission, the care burden often resides on the shoulders of families. Dovel and Thomson [23, 24] also pointed out financial problems and difficulty in affording medications. Wamoyi et al. [25] stated that most patients experienced a reduction in libido over the first 3–6 months of starting treatment owing to the fear of disease transmission and the strong advice of health consultants to follow health recommendations.

Labeling, including self‐stigma, criminalization and judgment and blaming and humiliation, was among the issues experienced by our patients. This observation agreed with the results of Safarzadeh Jahromy et al. [13, 15]. Accordingly, wrong judgment, stigmatization, discrimination, and social rejection are among important issues. Likewise, Ida Nur [26] stated that self‐stigmatization was likely to occur at the time of diagnosis, predisposed by the wrong judgments of health workers and people. Stutterheim et al. [27] highlighted patients’ experiences concerning stigmatization as humiliating reactions of others, keeping a physical distance, excessive precautions, social avoidance, abandonment, rejection, judgment, reprimanding, labeling, starting rumors, denial, and secrecy. Mahamboro et al. [28] further noted that PLHIV perceived external stigma in healthcare centers, society, and the family, in forms such as discriminatory attitudes and behaviors. Labeling and stigma, including self‐stigmatization, criminalization, and humiliation, further reduce motivation and compromise behavioral skills by creating fear of disclosure, social rejection, and avoidance of care [13, 15, 26, 27]. Addressing stigma is thus critical to enhancing both motivation and the practical skills required for treatment adherence.

Other experiences of patients included poor quality and insufficient medical services, inappropriate behavior with the patient and family, lack of respect for patient privacy, discrimination in medical services, and avoidance of providing services. This was in line with the results of Senyurek et al. [29] who addressed patient interactions with the health system and embedded the existing shortcomings such as lack of confidentiality of patient information, negative attitudes of some doctors toward the disease, and discrimination. Mahamboro et al. [28] also denoted external stigma received from health workers; Moradi et al. [30] pointed out the incomplete coverage of healthcare services, patient dissatisfaction with some care services provided by consultation centers/clinics, nonadherence to confidentiality, and lack of access to specialized care services.

As illustrated in the conceptual model (Figure 1), these themes are closely interconnected and operate as a reinforcing cycle. Emotional and psychological tensions form the core experience of living with the disease and contribute to behavioral responses such as social withdrawal, substance use, and poor treatment adherence. These responses, together with chronic illness, lead to social deprivation, including occupational instability and threatened marital and social relationships. Structural factors, particularly unfavorable social policies and inadequate medical services, intensify these difficulties by limiting access to financial, legal, and specialized support. Over time, these pressures result in profound life changes, including altered self‐perception, family disruption, and socioeconomic decline. Labeling and stigma cut across all themes, exacerbating psychological distress, discouraging care‐seeking, and reinforcing social exclusion. Overall, applying the IMB framework demonstrates that enhancing information, motivation, and behavioral skills is essential to improve adherence, psychosocial well‐being, and quality of life for PLHIV in Iran. This theoretical linkage strengthens the interpretation of our findings and provides a roadmap for developing culturally and contextually relevant interventions. Based on the findings, it can be noted the importance of designing and implementing targeted programs to increase awareness in the community in order to combat misinformation and reduce stigma, integrating mental health services into HIV care programs, training health workers in confidentiality, empathetic communication, and nondiscriminatory care, and expanding financial and social support for PLHIV and recognizing them as a vulnerable group. This study provides novel insights into the experiences of PLHIV in Iran, highlighting context‐specific social‐, cultural‐, and treatment‐related challenges that have not been thoroughly explored in previous research.

## 5. Conclusions

According to this study’s findings, PLHIV and their caregivers face numerous challenges and issues encompassing all the personal, familial, and social dimensions of their lives. Despite the fact that the diagnosis of the first case of this disease dates back many years, many people in society and even health workers have retained their negative attitudes toward this disease, imposing extra pressure on patients and their caregivers. Because there is no definitive treatment for the disease and regarding the identification of new cases, it is necessary for policymakers and healthcare officials to narrow their focus on this disease, PLHIV, and their caregivers. There is a need to treat physical problems and symptoms, as well as the psychological, familial, and social problems of patients, to increase their quality of life. Also, by boosting social awareness and properly informing people through mass media, it is possible to refine the negative attitudes of society and prevent social rejection, stigmatization, and discrimination against PLHIV. This would also help curb disease propagation due to nondisclosure.

### 5.1. Limitations

The most important limitations of this study included the sample size, limiting the generalizability of our findings to the entire community. Another limitation of this research included the participants′ concerns about the confidentiality of their information and fear of stigmatization. This problem even caused some people to refuse to attend the interview at the scheduled time. Prior to the interviews, participants were assured that their identifications, including their names and surnames, would not be recorded. Another limitation of the study is that it was conducted in one city (Kerman), which may limit transferability to other regions of Iran. Also, selecting participants from a counseling center and a positive club may have led to the inclusion of individuals who are more engaged in care, whereas those not linked to care may face even greater challenges.

## Author Contributions

Conceptualization: Asma Baniasad and Ghoncheh Raheb; methodology: Asma Baniasad, Ghoncheh Raheb, and Kian Norouzi Tabrizi; analysis: Asma Baniasad, Ghoncheh Raheb, and Maryam Latifian; research and review of sources: Asma Baniasad; drafting: Asma Baniasad, Ghoncheh Raheb, and Maryam Latifian; editing and finalization written by: Asma Baniasad and Maryam Latifian; supervision: Ghoncheh Raheb, Kian Norouzi Tabrizi, and Maryam Latifian.

## Funding

No funding was received.

## Ethics Statement

All procedures performed in studies involving human participants were under the ethical standards of the Institutional and National Research Committees and with the 1964 Declaration of Helsinki and its subsequent amendments or similar ethical standards. The present research is a part of a doctoral thesis with the code of ethics IR.USWR.REC.1399.210 approved by the University of Social Welfare and Rehabilitation Sciences. Participation in the study was voluntary, and all participants provided written informed consent. In this study, to protect the information and privacy of the participants, a numerical code was given to each of the participants and their names were not published anywhere.

## Consent

Please see the Ethics Statement.

## Conflicts of Interest

The authors declare no conflicts of interest.

## Data Availability

The datasets used and/or analyzed during the current study are available from the corresponding author on reasonable request.
